# From cultural constraints to structural resilience: a comparative analysis of end-of-life care policies in China, South Korea, the U.S., and the U.K.

**DOI:** 10.3389/fpubh.2026.1756405

**Published:** 2026-03-03

**Authors:** Songwu Luo, Dongje Cho, Jaehyun Cho

**Affiliations:** 1 Department of Law, Dong-A University, Busan, Republic of Korea; 2 Korean Constitutional Law Association, Seoul, Republic of Korea

**Keywords:** advance directives, comparative health policy, confucian culture, end-of-life care, palliative care policy, structural resilience

## Abstract

**Introduction:**

As global populations age at unprecedented rates, nations worldwide confront the challenge of developing effective end-of-life (EoL) care systems. While advance directives (ADs) represent a cornerstone of patient-centered dying, their adoption varies dramatically across jurisdictions. This study introduces a novel dual-dimensional analytical framework—“Resilience from Scale” and “Resilience from Structure”—to explain cross-national variations in EoL care policy effectiveness among China, South Korea, the United States, and the United Kingdom.

**Methods:**

Using a comparative, secondary-data-based design, we apply a Most Similar Systems Design to the East Asian cases and comparative institutional analysis to the Western cases. We employ semi-quantitative, ordinal coding of “Scale” and “Structure” indicators to assess how resource capacity and institutional configurations align with divergent policy outcomes.

**Results:**

The comparative evidence suggests that higher structural resilience—operationalized through national legislation, insurance integration, and state capacity/legitimacy—tends to be more consistently aligned with higher AD implementation than cultural explanations or resource availability alone. South Korea’s post-2018 shift following the Life-Sustaining Treatment Decision Act provides a salient within-case contrast, supporting the comparative plausibility that institutional design can coincide with substantial changes in advance care planning uptake even where cultural constraints are often assumed to be strong.

**Conclusion:**

These findings challenge culturally deterministic interpretations in EoL policy research and propose a “Structural Adaptation Model” as a heuristic for jurisdictions seeking to develop effective advance care planning systems.

## 1 Introduction

### 1.1 Background

The 21st century has ushered in an unprecedented demographic transformation (1). According to the World Health Organization, the global population aged 60 years and over will double from 12 to 22% between 2015 and 2050, with the absolute number of older adults projected to reach 2.1 billion (2). This “silver tsunami” carries profound implications for healthcare systems worldwide (3), particularly regarding how societies manage death and dying. The Economist Intelligence Unit’s Quality of Death Index—the most comprehensive global assessment of end-of-life care quality—reveals striking disparities: while the United Kingdom ranks first with a score of 93.9, China languishes at 71st with merely 23.3 points (4). Such variations are difficult to attribute solely to economic development, as nations with comparable resources exhibit vastly different outcomes (5).

Advance directives (ADs)—legal documents enabling individuals to articulate their healthcare preferences before losing decision-making capacity—have emerged as a central mechanism for ensuring patient-centered dying (6). The World Health Organization has designated palliative care integration into national health systems as a priority, emphasizing that “access to palliative care is a human right (7).” Yet despite this global consensus, the implementation of advance care planning varies dramatically (8). In the United States, approximately 36.7% of adults have completed any form of advance directive (9). In the United Kingdom, the ReSPECT process has systematically integrated advance care planning into routine clinical practice. In stark contrast, China—home to over 209 million older adults—had fewer than 50,000 registered living wills by 2022 (10), representing a mere 0.005% of the adult population (11).

### 1.2 The research puzzle

This study addresses two interrelated puzzles that challenge conventional explanations of end-of-life care policy development. First, why do China and South Korea—nations sharing deep Confucian cultural roots (12), comparable death taboos, and family-centered decision-making traditions—exhibit such dramatic divergence in AD adoption? South Korea’s National Agency for Management of Life-Sustaining Treatment reports that cumulative AD registrations surged from fewer than 100,000 in 2018 to over 3 million by August 2025, representing a 30-fold increase within 7 years (13, 14). Meanwhile, China’s registration numbers remain negligible despite possessing the world’s largest aging population. This “temporal gap” and “efficacy gap” between culturally similar nations suggests that culture alone cannot explain policy outcomes (15).

A substantial body of end-of-life scholarship attributes the limited uptake of advance care planning in many Asian and family-centric settings primarily to cultural norms—most notably death taboo, relational autonomy, and Confucian filial obligations that privilege family-centered decision-making over individual preference articulation (16). Qualitative and review evidence has repeatedly highlighted collusion, indirect disclosure practices, and the moral pressure to pursue life-prolonging treatment as culturally grounded barriers to formalized advance directives (15, 17).

While these cultural accounts capture important micro-level constraints on communication and decision-making, they often remain mechanism-light at the policy level and provide limited leverage for explaining rapid within-society change or sharp divergence among culturally proximate systems. In particular, culture-centered explanations struggle to account for (i) the magnitude and timing of South Korea’s post-2018 adoption shift, and (ii) the persistent gap between the United States and the United Kingdom despite broadly shared Anglo-American normative commitments. These puzzles motivate a complementary institutional perspective: whether differences in legal authority, financing integration, governance capacity, and information infrastructure condition how cultural preferences are translated into implementable end-of-life policy.

Second, why does the United States—possessing unparalleled healthcare resources with per capita health expenditure exceeding $14,000 annually (18)—struggle with over treatment, late hospice referrals, and fragmented care coordination (19)? Over 35% of Medicare decedents die within one week of hospice enrollment (20), suggesting that resource abundance does not automatically translate into quality dying. Conversely, the United Kingdom, with comparatively modest per capita spending of approximately $6,500, consistently achieves the highest Quality of Death rankings globally (21). This paradox is consistent with the view that the relationship between resources and outcomes is mediated by institutional factors.

### 1.3 Objectives and contribution

In this study, we use end-of-life care system resilience to denote a health system’s capacity to sustain and improve preference-concordant end-of-life care under chronic demographic pressure, drawing on health-system resilience scholarship that emphasises absorptive, adaptive, and transformative capacities (22).

We operationalise this concept through two analytically distinct dimensions. Resilience from Scale captures the absorptive (buffer) capacity—the level of material resources and service capacity available relative to the magnitude of ageing-related demand. Resilience from Structure captures the institutional conversion capacity—the governance, legal authority, financing arrangements, and information infrastructure that translate available resources into accessible, coordinated, and preference-concordant services (23).

This decomposition is necessary because resource abundance is a prerequisite but not a guarantee of effective end-of-life care; cross-national divergence is expected when institutional configurations generate different “conversion efficiencies” from comparable resource bases.

By deploying this framework across four strategically selected cases, we pursue three objectives. First, we examine whether structural resilience provides a more plausible explanation of cross-national variation in AD implementation than cultural predisposition or resource availability alone. Second, we challenge prevailing “cultural determinism” that portrays Confucian societies as inherently resistant to advance care planning, using South Korea’s transformation as a revealing case. Third, we propose a “Structural Adaptation Model” that synthesises actionable policy implications for countries seeking to build effective end-of-life care systems.

Our contribution is threefold. Theoretically, we move beyond the culture-versus-resources dichotomy by specifying institutional configuration as a mediating mechanism. Methodologically, we employ a Most Similar Systems Design to structure cross-case comparison and make explicit the assumptions under which institutional contrasts are interpreted. Practically, we distill lessons from South Korea’s structural transformation that may inform China’s ongoing policy development and guide other countries navigating similar transitions.

Finally, the study is designed as theory-building comparative analysis. Accordingly, the claims advanced here are framed in terms of comparative plausibility and cross-case pattern consistency rather than universal causal laws. The framework is intended to identify which configurations are most consistent with observed divergence across strategically selected cases, while recognizing that causal identification would require additional designs and data beyond the scope of this study.

## 2 Materials and methods

### 2.1 Research framework

This study operationalizes end-of-life care system performance through a dual-dimensional framework that disaggregates “resilience” into complementary components. We define Resilience from Scale as the healthcare system’s “hard power” capacity to absorb mortality-related demands generated by population aging. This dimension captures the raw resource base available for end-of-life care provision, independent of how efficiently such resources are deployed. Key indicators include: total and per capita gross domestic product (GDP), per capita health expenditure, palliative care beds per million population, and the absolute number of practicing physicians.

We define Resilience from Structure as the “soft power” institutional efficiency that transforms available resources into effective end-of-life services. This dimension captures the policy architecture, legal frameworks, and cultural adaptation mechanisms that mediate between resource inputs and care outcomes. Key indicators include: the level and comprehensiveness of national legislation (presence of dedicated end-of-life care statutes), payment system configuration (national health insurance coverage versus commercial insurance dependence), information infrastructure (existence of national AD registration platforms), and cultural adaptation mechanisms (legal provisions for family proxy decision-making that reconcile individual autonomy with collectivist values).

**Figure 1:**
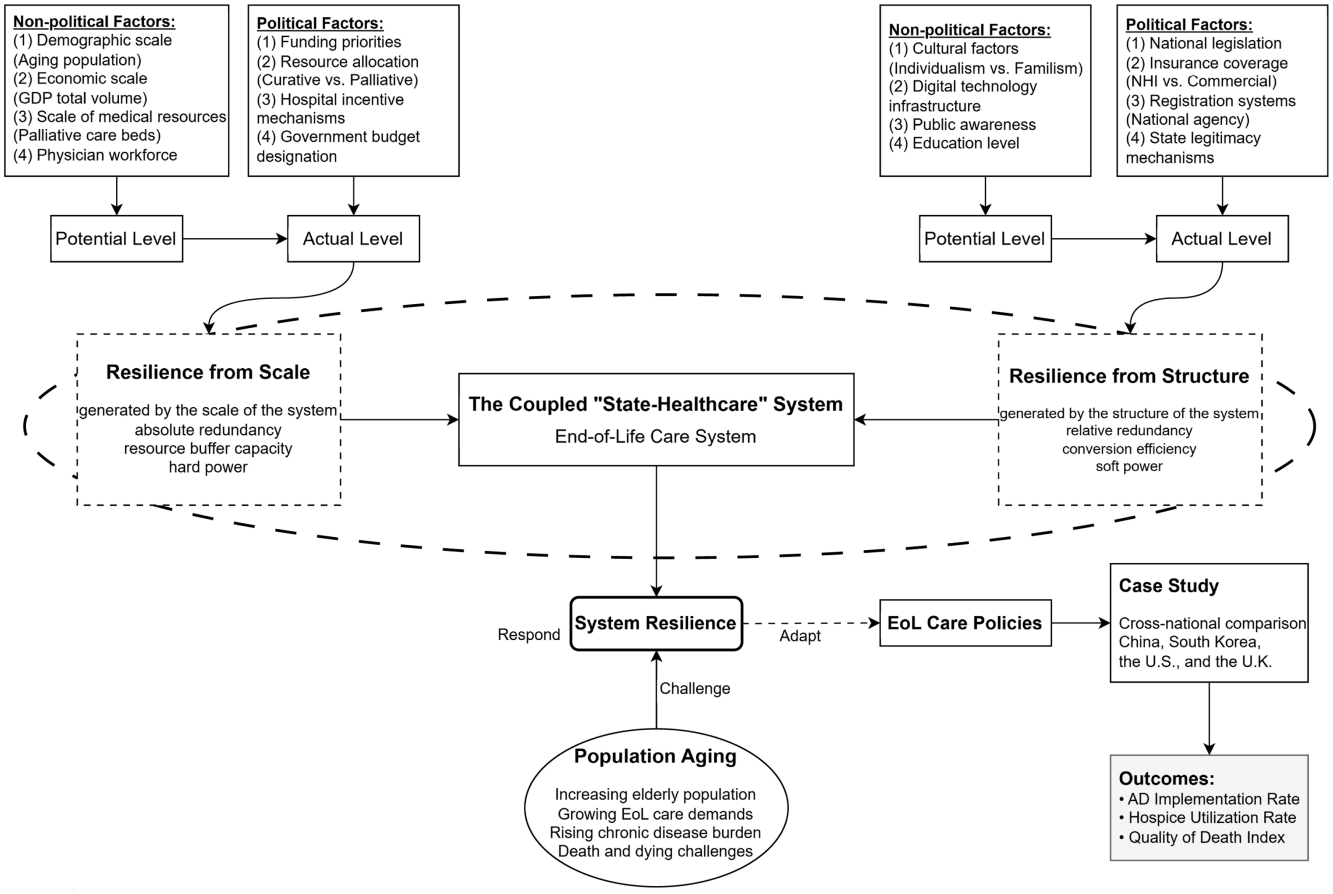
Analytical framework of end-of-life care resilience. The figure depicts a flow diagram with two input streams converging upon the end-of-life care system. The left stream represents “Resilience from Scale” (input factors: GDP/economic capacity, health expenditure, palliative care beds, physician workforce). The right stream represents “Resilience from Structure” (input factors: national legislation, insurance coverage, registration systems, cultural adaptation mechanisms). These inputs are processed through the central “EoL care system” transformation box, producing outputs measured as AD implementation rate, hospice utilization rate, and quality of death index scores. Arrows indicate that both scale and structure must operate synergistically; scale provides the resource substrate while structure determines conversion efficiency.

The analytical logic proceeds as follows: Scale provides the necessary foundation—without adequate resources, no amount of institutional sophistication can deliver quality care. However, Scale alone is insufficient; resources must be channeled through effective institutional conduits to reach patients. Structure determines the conversion efficiency, explaining why nations with comparable resource bases achieve divergent outcomes. Figure 1 illustrates this conceptual model.

### 2.2 Case selection and methodology

This study employs a strategic case selection approach combining Most Similar Systems Design (MSSD) with cross-cultural comparative analysis. Four nations—China, South Korea, the United States, and the United Kingdom—were selected based on theoretical rather than convenience criteria, enabling both within-group and across-group comparisons that illuminate the independent effects of institutional variables.

Case selection criteria: Case selection was theory-driven and guided by four explicit criteria. First, we sought maximal outcome variation in advance directive (AD) uptake and end-of-life (EoL) system performance, including a low-uptake case (China), a rapid institutional take-off case (South Korea), a high-resource but coordination-challenged case (United States), and a high-performing benchmark case (United Kingdom). Second, we selected cases to provide planned contrasts on the two dimensions of the proposed framework—variation in resource capacity (Scale) and variation in institutional conversion capacity (Structure)—so that institutional mechanisms could be examined without treating “resources” or “culture” as monolithic explanations. Third, we prioritized design leverage through nested paired comparisons (within-region and cross-region), enabling both controlled within-pair inference and broader cross-pair plausibility checks. Fourth, cases were required to have sufficiently transparent secondary data availability (national registries, official statistics, and internationally harmonized databases) to support reproducible cross-national comparison.

These criteria motivate the nested paired comparisons described below.

Supplementary Table S1 reports the ordinal coding rubric used for cross-national classification, while Supplementary Table S2 summarizes the MSSD matching conditions and rival-explanation checks that structure the paired comparisons.

The East Asian pairing: China and South Korea: These nations constitute an ideal Most Similar Systems configuration for isolating institutional effects. Both share profound Confucian cultural heritage emphasizing filial piety, family-centered decision-making, and traditional death taboos. Historically, both societies treated death discussions as inauspicious and delegated end-of-life decisions to family patriarchs rather than individual patients. Both experienced rapid economic development, transitioning from agrarian to industrial economies within a generation. Both possess high-quality digital infrastructure capable of supporting national registration systems. By controlling for these cultural and technological similarities, observed differences in AD adoption can be attributed to the institutional variables that diverge: South Korea’s comprehensive 2018 legislation and full National Health Insurance integration versus China’s fragmented local regulations and absent insurance coverage.

The Western pairing: the United States and the United Kingdom: These nations represent mature end-of-life care systems within the Western individualist tradition, yet exemplify contrasting structural configurations. The United States operates a market-dominated healthcare system where commercial insurance, fee-for-service payment models, and defensive medicine practices shape end-of-life care delivery. The 1990 Patient Self-Determination Act established federal requirements for AD documentation, yet implementation remains fragmented across 50 states with varying legal standards. The United Kingdom operates a unified National Health Service (NHS) system where general practitioners serve as gatekeepers, the Gold Standards Framework provides standardized care pathways, and hospice funding integrates government and charitable sources. This pairing illuminates how equivalent cultural acceptance of patient autonomy produces divergent outcomes under different structural regimes.

Cross-group comparisons: Comparing across the East Asian and Western pairings enables assessment of whether cultural values constitute absolute barriers or malleable constraints. If South Korea—despite its Confucian heritage—achieves AD implementation rates approaching Western levels following structural intervention, this is consistent with the comparative proposition that institutional design can coincide with meaningful shifts in uptake under culturally constraining conditions. Conversely, if the United States—despite its individualist culture—underperforms relative to the United Kingdom under fragmentation, this suggests that favorable cultural predispositions may be insufficient without coherent institutional support.

Analytical leverage of the four-case set: Taken together, these cases approximate a two-dimensional comparative space implied by the framework. China represents a setting where demographic pressure is large but institutional conversion mechanisms remain underdeveloped; South Korea represents a setting where major institutional reforms created high conversion capacity under similar Confucian-cultural constraints; the United States represents a high-resource system in which fragmentation and incentive misalignment can weaken conversion; and the United Kingdom provides a benchmark of comparatively coherent institutional arrangements under moderate resources. This four-case configuration enables us to evaluate whether cross-national divergence is more consistent with differences in institutional conversion capacity (Structure) than with culture or resource abundance alone, while remaining explicit that the evidence supports comparative plausibility rather than definitive causal identification.

Consistent with this design and its emphasis on cross-national transparency and comparability, data sources include official government statistics (National Health Commission of China, Korean Ministry of Health and Welfare, U.S. Centers for Medicare and Medicaid Services, NHS England), international databases (OECD Health Statistics, World Bank Development Indicators, WHO Global Health Observatory), peer-reviewed literature, and specialized registries (Korean National Agency for Management of Life-Sustaining Treatment, U.S National Hospice and Palliative Care Organization). The temporal scope focuses on 2019–2024 data, capturing both pre- and post-pandemic conditions while emphasizing the most recent available statistics.

Implementing the most similar systems design (MSSD): We operationalize MSSD through structured within-pair comparisons and explicit rival-explanation checks. For the East Asian pair (China–South Korea), we treat Confucian family-centered norms, rapid late-industrialization trajectories, and high digital readiness as matching conditions, while treating national legal authority, insurance/payment alignment, and state legitimation/registry governance as the key institutional contrasts of interest. For the Western pair (United States–United Kingdom), we treat high-income status, liberal-individualist value orientation, and mature end-of-life care sectors as matching conditions, while leveraging variation in health-system financing/coordination and legal-institutional coherence as the key contrasts.

Rival explanations and structured comparison protocol: To reduce *post hoc* interpretation, we pre-specify three rival explanations—(i) cultural determinism, (ii) resource abundance (Scale) as a sufficient driver of adoption and performance, and (iii) technology/digital readiness as a sufficient driver—and evaluate them using nested paired comparisons. If culture were determinative, China and South Korea should display similarly low adoption under shared Confucian constraints; if resources were sufficient, the United States should dominate outcomes given its resource profile; if technology were sufficient, high digital readiness would translate into uniformly high adoption. The observed divergence across the paired contrasts is therefore interpreted as more consistent with institutional “conversion capacity” (Structure) than with culture, resources, or technology alone, while acknowledging that the evidence supports comparative plausibility rather than definitive causal identification.

Clarifying MSSD assumptions and governance differences. While China and South Korea differ in broader political governance arrangements, these differences are not treated as uncontrolled background noise but are analytically subsumed within the institutional contrasts of legal authority, insurance alignment, and state legitimation mechanisms. Because regime type shapes exactly the institutional channels emphasized by our framework—legal authority, payment/insurance coordination, and state legitimation—it is treated as a constitutive element of “Structure” rather than an exogenous confounder to be controlled away. The MSSD assumption in this study therefore does not require identical regime types, but functional similarity in the social, cultural, and developmental conditions under which end-of-life institutions operate. By holding constant Confucian family norms, late-industrialization trajectories, and digital capacity, and by explicitly modeling governance-related variation as part of institutional “Structure,” the comparative design isolates how different modes of state coordination and legitimation translate policy intent into clinical practice. Accordingly, governance differences are treated as explanatory variation rather than as confounding factors that undermine comparative inference.

### 2.3 Definitions and factors influencing two types of resilience

#### 2.3.1 Resilience from scale

Resilience from Scale represents the healthcare system’s capacity to absorb the mortality demands generated by population aging—what we term the “resource buffer against the aging tsunami.” This dimension captures whether a nation possesses sufficient raw materials—financial resources, physical infrastructure, and human capital—to potentially deliver adequate end-of-life care. Critically, Scale measures potential capacity rather than actual deployment; a nation may possess abundant resources yet allocate them elsewhere due to political priorities or institutional incentives.

Demographic Scale measures the absolute magnitude of aging-related care demands. China’s older population (aged 65 and above) reached 216.76 million in 2023, representing 15.4% of the total population—the largest absolute number globally. The United States follows with approximately 61.2 million older adults (18% of population). South Korea crossed the “super-aged society” threshold of 20% older adults in December 2024 (24), achieving the fastest aging rate globally. The United Kingdom’s 12.7 million older adults (19% of population) represent a more moderate demographic challenge. These figures determine the denominator against which resources must be measured: larger older populations require proportionally greater Scale to maintain equivalent per-capita service levels.

Economic Scale encompasses both aggregate wealth and healthcare-specific investment. The United States leads decisively with per capita GDP exceeding $81,000 and per capita health expenditure approaching $14,000 (PPP)—more than double any other nation in this study. The United Kingdom follows with per capita GDP of approximately $48,900 and health expenditure around $6,500. South Korea’s per capita figures ($32,400 GDP; $4,400 health expenditure) reflect its developed economy status, while China’s per capita health expenditure of approximately $1,400—despite massive GDP growth—reveals the dilution effect of its 1.4 billion population. Importantly, China’s total health expenditure ranks second globally in absolute terms, creating potential Scale capacity that per capita figures obscure.

Medical Resource Scale focuses on palliative care-specific infrastructure and workforce. Palliative care beds per million population provide the most direct measure of end-of-life care capacity: the United Kingdom leads with approximately 50.8 beds per million in England (reaching 67.4 in Scotland), followed by South Korea with approximately 30 beds per million, while China’s figures remain below 5 beds per million despite recent expansion. Total physician workforce—approximately 4.2 million in China, 1.1 million in the United States, 320,000 in the United Kingdom, and 130,000 in South Korea—indicates the human capital base available for potential redeployment toward palliative specialization. The United States boasts the largest certified hospice and palliative medicine specialist workforce with nearly 20,000 board-certified physicians and 156 accredited fellowship programs.

Aggregating these indicators requires recognizing that absolute totals and per capita rates convey different information. China possesses enormous Scale in absolute terms—the largest older population, second-largest total health expenditure, and largest physician workforce—yet modest Scale in per capita terms given its population denominator. Conversely, South Korea and the United Kingdom possess moderate absolute Scale but higher per capita efficiency. The United States combines high absolute and per capita Scale, representing the maximum resource case. These baseline assessments, however, capture only potential capacity; actual resource deployment depends on political and institutional factors examined subsequently.

#### 2.3.2 Resilience from structure

Resilience from Structure represents the institutional efficiency with which available resources are converted into effective end-of-life services. Structure determines whether resources reach patients in appropriate forms and timely fashion—the “conversion coefficient” that transforms potential capacity into realized care. Four interrelated components constitute structural resilience.

Legislative Architecture refers to the legal framework governing end-of-life decision-making, particularly whether dedicated national legislation exists and its scope. The United Kingdom’s Mental Capacity Act 2005 provides comprehensive statutory grounding for Advance Decisions to Refuse Treatment, with clear requirements for validity and applicability. South Korea’s 2016 “Act on Hospice and Palliative Care and Decisions on Life-Sustaining Treatment” (effective 2018) represents the most comprehensive Asian legislation (25), establishing legal procedures for AD registration, institutional designation, and treatment withdrawal. The United States’ Patient Self-Determination Act (1990) mandates institutional policies but defers substantive regulation to states, creating a patchwork of 50 different legal regimes. China lacks national legislation entirely; Shenzhen’s 2023 local regulation represents the sole jurisdiction with statutory AD recognition. Legislative comprehensiveness determines whether patients and providers possess legal certainty regarding end-of-life decisions.

Payment System Configuration determines whether financial incentives facilitate or impede palliative care access. The United Kingdom’s NHS provides universal coverage with no patient cost-sharing for hospice services. South Korea’s National Health Insurance has covered inpatient hospice since 2015, with daily reimbursement rates exceeding $270 (354,497 KRW) that make hospice financially viable for providers. The United States’ Medicare hospice benefit—utilized by 49.1% of Medicare decedents in 2022—provides the most generous per-diem reimbursement globally (26), yet commercial insurance fragmentation and fee-for-service incentives in acute care create systematic biases toward aggressive treatment. China lacks specific health insurance codes for palliative care; services are reimbursed under general categories that fail to reflect true costs, rendering hospice economically unsustainable for hospitals operating under diagnosis-related group (DRG) payment systems.

Information Infrastructure encompasses national registration systems that ensure AD accessibility and portability. South Korea’s National Agency for Management of Life-Sustaining Treatment operates a centralized digital registry accessible to all designated medical institutions, enabling real-time verification of patient preferences. The United Kingdom’s ReSPECT process, though not a singular registry, provides standardized documentation integrated with electronic health records. The United States lacks a unified national registry; AD documentation remains fragmented across provider systems, often inaccessible during emergencies. China’s “Choice and Dignity” platform operates as a non-governmental initiative with no official recognition or clinical integration.

Cultural Adaptation Mechanisms refer to legal and procedural provisions that reconcile Western-originated autonomy principles with local value systems (27). Most critically, this involves how legislation addresses family proxy decision-making. South Korea’s law explicitly incorporates family consensus procedures, permitting collective family decisions when patients lack capacity—thereby accommodating Confucian family-centeredness within a legal autonomy framework. The law’s provision for state legitimacy through a National Agency effectively provides “official permission” that relieves families of moral stigma associated with treatment withdrawal. China’s Shenzhen regulation similarly permits family-based decisions but lacks national authority. Anglo-American systems, designed for individualist cultures, may require adaptation before transplantation to collectivist contexts.

### 2.4 Analytic strategy and coding rules

To reduce subjectivity in cross-national comparison, we implemented a rule-based, semi-quantitative coding strategy that translates heterogeneous secondary indicators into ordinal categories. “High/medium/low” labels are used as transparent ordinal positions in a two-dimensional comparative space rather than as precise measurements.

For each dimension, we (i) specified indicators ex ante (Supplementary Table S1), (ii) applied explicit inclusion criteria and coding thresholds to assign each indicator a 0–2 ordinal score (low = 0, medium = 1, high = 2), and (iii) aggregated indicator scores within each sub-dimension using a simple decision rule (majority/triangulation) to assign an overall category.

Indicator weighting. Within each sub-dimension, indicators are treated as equally weighted (each scored 0–2) and combined using the aggregation rules specified in Supplementary Table S1. Equal weighting is adopted to minimize subjective calibration in a theory-building, ordinal design and to preserve transparency and replicability across heterogeneous secondary data sources. As a limitation, alternative weighting schemes could shift borderline classifications; we therefore interpret all classifications as ordinal and heuristic rather than as precise quantitative estimates.

Indicators based on internationally harmonized quantitative data (e.g., OECD/WHO/World Bank statistics) are treated as formal measures; institutional features (e.g., legislation, payment design, registry infrastructure, state endorsement) are treated as rule-coded proxies with explicit criteria.

We report both “potential” levels (non-political baseline capacity/constraints) and “actual” levels (post-policy implementation), where the latter is derived by applying policy-implementation indicators (funding priorities and allocation for Scale; legislation, insurance/payment alignment, and state legitimation for Structure). Full coding rules, data-year alignment, and indicator roles are provided in Supplementary Table S1 to ensure reproducibility.

Worked coding example (South Korea, Actual Structure). To illustrate replication, we provide a step-by-step application of the Supplementary Table S1 rubric to South Korea’s Actual Structure classification. First, we coded Legislation as High (=2) because South Korea has comprehensive national end-of-life legislation with enforceable procedures (the Act on Hospice and Palliative Care and Decisions on Life-Sustaining Treatment, effective 2018). Second, we coded Payment Alignment as High (=2) because National Health Insurance explicitly reimburses hospice/palliative care with stable incentives (e.g., inpatient hospice reimbursement of 354,497 KRW/day). Third, we coded State Legitimation as High (=2) because a national agency (NALST) provides official endorsement and implementation authority via a centralized registry. We then applied the Supplementary Table S1 aggregation rule for Actual Structure by summing the three indicator scores (2 + 2 + 2 = 6); a total score of 5–6 maps to a High Actual Structure category.

Outcome hierarchy and proxy rationale. In this study, advance directive (AD) registration/coverage is treated as an intermediate implementation output—capturing policy reach, documentation visibility, and institutionalization—rather than as a direct measure of preference-concordant bedside decisions or patient experience. Whenever available, downstream outcome indicators (e.g., place-of-death patterns, hospice timing/utilization, and composite Quality-of-Death measures) are used to triangulate whether higher implementation plausibly corresponds to better end-of-life care. Because cross-nationally comparable measures of preference concordance and patient-reported end-of-life experience are not consistently available, we interpret registration differences ordinally and inferentially, and we avoid treating them as numerically equivalent quality differences across countries.

### 2.5 Data comparability and year alignment

Because this study synthesizes secondary indicators generated under different national administrative, legal, and clinical definitions, we adopt an explicit protocol to address cross-national comparability. We prioritize internationally harmonized series (OECD, WHO, and World Bank) when available and treat them as formal quantitative measures. Country-specific administrative counts (e.g., registry-based advance directive registrations and hospice/palliative service capacity reported by national agencies or professional bodies) are treated as rule-coded indicators (i.e., proxies), and their definitional scope and data provenance are documented in Supplementary Table S1.

To address time-frame mismatch, we apply a year-alignment rule: for each indicator, we use the most recent observation available within the study window (2019–2024) and, where feasible, align countries around a common reference period (typically 2021–2023). When exact alignment is not feasible, we record the country-specific year in Supplementary Table S1 and interpret the evidence ordinally rather than as exact point estimates.

Finally, we distinguish indicators that are conceptually and operationally comparable across countries (e.g., per-capita health expenditure in PPP terms) from those that are only partially comparable because national definitions and reporting practices differ (e.g., the scope of “hospice services,” “palliative care beds,” and the operational meaning of “advance directives”). For partially comparable indicators, inference relies on triangulation across multiple sources and on ordinal placement (low/medium/high) rather than on assumptions of numerical equivalence.

Accordingly, the purpose of the comparative analysis is not to establish exact cross-sectional equivalence across countries, but to assess structured comparative plausibility under explicitly stated assumptions and constraints. All inferences are therefore design-consistent with the ordinal coding strategy and the Most Similar Systems framework rather than dependent on precise numerical equivalence.

Methodological status of the framework. This study adopts a semi-quantitative comparative framework designed for theory-building rather than causal estimation. “Scale” and “Structure” are treated as analytical dimensions that organize cross-national variation, not as directly measurable variables. Indicators associated with each dimension are therefore assigned different analytical roles: internationally harmonized statistics are treated as formal measures, while country-specific administrative data function as rule-coded indicators (proxies) supporting ordinal classification. The resulting typology is not intended as a *post hoc* classificatory list, but as a design-consistent heuristic model that enables structured comparative plausibility assessment under explicitly stated assumptions. The analytical status of each indicator and its role within the comparative framework are summarized in Supplementary Table S3.

## 3 Results

Outcome hierarchy note. Throughout the Results section, AD registration/coverage is reported as an intermediate implementation output (policy reach and institutionalization) and is interpreted alongside downstream outcome indicators (e.g., hospice timing/utilization, place-of-death patterns, and Quality-of-Death measures) rather than as a direct measure of preference-concordant bedside care.

### 3.1 Differences in two types of resilience between four countries

#### 3.1.1 Differences in resilience from scale

Non-political factors and potential levels: Assessment of Scale resilience begins with objective, non-political indicators that establish baseline resource capacity. These “hard” measures—demographic burden, economic capacity, and medical infrastructure—exist independently of policy choices, representing the raw material from which end-of-life care systems must be constructed.

Demographic analysis reveals that China and the United States confront aging challenges of fundamentally different magnitude than South Korea and the United Kingdom (28). China’s 216.76 million older adults represent a population larger than most nations’ total inhabitants; even modest per capita improvements require astronomical absolute investments. The United States’ 61.2 million older adults, while substantially smaller, still constitute a large absolute population requiring extensive service infrastructure. South Korea’s 10.24 million and the United Kingdom’s 12.7 million older adults populations, though representing higher proportions of their total populations (20 and 19% respectively), constitute smaller absolute numbers.

Economic indicators show the United States at the highest Scale level. Per capita health expenditure of approximately $14,000 (PPP) exceeds China’s $1,400 by a factor of 10 and surpasses the United Kingdom’s $6,500 by more than double. Total health expenditure as a share of GDP—17% for the United States compared to 11% for the United Kingdom, 9.6% for South Korea, and 7% for China—reflects these cross-national differences. However, China’s aggregate health expenditure ranks second globally in absolute terms, creating substantial total Scale despite low per capita figures.

Medical resource indicators reveal similar patterns. The United Kingdom leads in palliative care beds per million population (50.8 in England, 67.4 in Scotland) (29), followed by South Korea (approximately 30), while China’s ratio remains below 5 despite hosting 510 hospitals with hospice departments by 2020. Physician workforce totals—4.2 million in China, 1.1 million in the United States, 320,000 in the United Kingdom, 130,000 in South Korea—indicate China’s absolute resource advantage and South Korea’s relative constraint. The United States’ 19,920 board-certified hospice and palliative medicine specialists represent a specialized workforce unmatched elsewhere Table 1.

**Table 1:** Potential levels of resilience from scale (resource capacity).

Country	Demographic scale (Aging population)	Economic scale (GDP/Health spending)	Medical resource scale (Beds/Physicians)	Potential level
China	High (216.76 M older adults; 15.4%)	High total volume; Low per capita ($1,400)	High total (4.2 M physicians); Low per capita beds (<5/million)	High
South Korea	Medium (10.24 M older adults; 20%)	Medium ($32,400 GDP; $4,400 health)	Medium (130 K physicians; 30 beds/million)	Medium
United States	High (61.2 M older adults; 18%)	High ($81,695 GDP; $14,000 health)	High (1.1 M physicians; 19,920 PC specialists)	High
United Kingdom	Medium (12.7 M older adults; 19%)	Medium ($48,912 GDP; $6,500 health)	Medium (320 K physicians; 50.8 beds/million)	Medium

Coding rules and thresholds for all high/medium/low classifications are reported in Supplementary Table S1.

Political factors and actual levels: Raw resource indicators, however, provide misleading assessments of actual end-of-life care capacity. The translation of potential resources into realized services depends critically on political will and funding priorities. Resources “possessed” are not equivalent to resources “deployed”—a distinction that fundamentally alters Scale assessments.

China exemplifies the gap between potential and actual Scale. Despite enormous aggregate resources, public hospital incentive structures systematically divert capacity away from palliative care. Performance evaluation metrics emphasizing surgical volumes, ICU utilization, and revenue generation render hospice services financially unattractive. Under diagnosis-related group (DRG) payment systems, palliative care generates minimal reimbursement while occupying beds that could host more lucrative procedures. Consequently, less than 7% of Chinese patients requiring hospice care currently receive it. China’s actual Scale resilience falls substantially below its potential level as indicated in Table 2.

**Table 2:** Actual levels of resilience from scale (modified by allocation policy).

Country	Impact of funding priorities	Impact of resource allocation (curative vs. palliative)	Actual level
China	Low priority; No dedicated budget lines for palliative care	Heavily skewed toward curative/acute care (ICU, surgery); minimal fiscal support for hospice	Lower than potential
South Korea	High priority post-2018; Designated hospice expansion budgets	Partial skew toward curative hospital incentives, but targeted palliative support through government-mandated quotas and NHI reimbursement that helps sustain hospice services	Medium (Consistent)
United States	High spending overall; $23.7B Medicare hospice (2022)	High though inefficient; absolute abundance despite waste	High (Consistent)
United Kingdom	General NHS funding supplemented by substantial charitable contributions; Limited dedicated hospice expansion budgets	Strong integration; high resource utilization efficiency	Medium (Consistent)

“Actual” Scale levels are derived from policy-implementation indicators following the coding rubric in Supplementary Table S1.

South Korea shows the opposite trajectory. Following the 2018 legislation, the government established dedicated budget lines for hospice expansion, designated specific institutional quotas for palliative care beds, and created financial incentives for hospice program development. Official statistics show that designated hospice institutions (all types combined) expanded from 54 in 2013 to 181 by 2022, with total designated beds reaching 1,601 in 2022 (30). National Health Insurance coverage, providing daily reimbursement rates that ensure institutional viability, transformed hospice from a charitable endeavor to a financially sustainable service line. South Korea’s actual Scale resilience aligns with its potential level, corresponding with these policy interventions.

The United States maintains consistent high Scale given its sheer resource abundance. Medicare hospice spending exceeded $23.7 billion in 2022 (31), with 5,899 certified hospice programs and 49.1% of Medicare decedents utilizing hospice services. Absolute resource availability ensures that Scale constraints rarely limit access for insured populations. The United Kingdom similarly maintains actual Scale at its above potential levels through the combination of NHS funding and charitable contributions. UK hospices require approximately £2 billion annually; while government provides only £500 million directly, the combination of statutory and voluntary sector resources ensures adequate capacity.

#### 3.1.2 Differences in resilience from structure

Non-political factors and potential levels: Structural resilience assessment begins with socio-cultural foundations that exist prior to policy intervention. These “soft” environmental factors—cultural attitudes toward death and autonomy, digital infrastructure, and public education—establish baseline conditions that legislation must either leverage or overcome.

Cultural orientation toward death and decision-making constitutes a central non-political factor. China and South Korea share deep Confucian traditions that fundamentally shape end-of-life contexts. The concept of filial piety creates powerful obligations for adult children to preserve parental life through all possible means (32); authorizing treatment withdrawal may be perceived as abandoning one’s parents—a profound moral failure with social consequences. Death itself remains taboo; discussing mortality is considered inauspicious, discouraging advance care conversations. Family-centered decision-making traditions position the collective unit rather than the individual patient as the legitimate authority. These cultural factors can create baseline resistance under prevailing norms to Western-style advance directives premised on individual autonomy and explicit death planning (33).

Anglo-American cultures present fundamentally different orientations. The philosophical tradition of individual autonomy, codified in bioethical principles since Beauchamp and Childress, presumes that competent adults possess the right to determine their own medical fates. The “death positive” movement, particularly prominent in the United Kingdom, actively destigmatizes mortality discussions. While family involvement remains important, legal systems recognize individual patients as primary decision-makers. These cultural predispositions provide favorable baseline conditions for advance directive acceptance without requiring extensive adaptation.

Digital infrastructure and educational attainment represent enabling factors that facilitate implementation once cultural barriers are addressed. All four nations possess robust digital infrastructure capable of supporting national registration systems; internet penetration exceeds 70% in each country, with South Korea and China leading in mobile connectivity. Educational attainment—affecting citizens’ capacity to comprehend AD documentation and engage in advance care planning—ranges from moderate in China to high in the other three nations (34). However, these enabling factors cannot overcome cultural resistance independently; they determine implementation feasibility once cultural barriers are addressed through other means (Table 3).

**Table 3:** Potential levels of resilience from structure (socio-cultural foundation).

Country	Cultural factors (individualism vs. familism)	Digital infrastructure	Public awareness/Education	Potential level
China	Low (Strong death taboo; family-centered; Confucian filial piety)	High	Medium	Low
South Korea	Low (Confucian tradition; strong filial obligations)	High	High	Low/Medium
United States	High (Individual autonomy culture; self-determination)	High	High	High
United Kingdom	High (Death positive movement; autonomy tradition)	High	High	High

See Supplementary Table S1 for coding rules.

Political factors and actual levels: Political institutions—including legislation, insurance integration, and state agencies—shape actual structural resilience levels. The following observations document how these institutional factors correspond with divergent outcomes across the four countries.

South Korea underwent substantial structural transformation following the 2018 implementation of the “Act on Hospice and Palliative Care and Decisions on Life-Sustaining Treatment.” This legislation created comprehensive legal architecture that corresponded with elevated structural resilience despite unfavorable cultural baselines. Three institutional mechanisms characterize this transformation. First, the law established clear legal procedures for AD registration and treatment withdrawal, providing both patients and physicians with legal certainty that had previously been absent—recall that the 1997 Boramae Hospital case resulted in physician convictions for following family withdrawal requests. Second, National Health Insurance integration ensured that palliative care was financially accessible; the daily reimbursement rate of 354,497 KRW (approximately $270) for inpatient hospice removed economic barriers for patients while ensuring institutional viability. Third, the establishment of the National Agency for Management of Life-Sustaining Treatment created government endorsement that confers official status on end-of-life decisions. For adult children concerned about social stigma associated with “abandoning” parents, the existence of official government registration and authorization provides institutional support; decisions made through state-sanctioned procedures carry official documentation.

China, despite sharing South Korea’s Confucian heritage, lacks equivalent institutional intervention. No national legislation governs advance directives; Shenzhen’s 2023 regulation remains a lone local experiment without replication elsewhere. No specific health insurance codes exist for palliative care; services are reimbursed under general categories that fail to reflect true costs. No national registration system provides legal verification or state authorization. In this context, cultural barriers to advance care planning remain unaddressed by institutional countervailing forces; death discussions remain taboo, family decision-making remains legally ambiguous, and the mere 50,000 registered living wills represent negligible uptake. China’s actual structural resilience remains at low levels consistent with its cultural baseline.

The United States presents a case where favorable cultural conditions coexist with structural fragmentation. Despite cultural acceptance of individual autonomy and three decades since the Patient Self-Determination Act, actual structural resilience falls below potential levels in the context of institutional incoherence. Fifty different state legal frameworks create compliance complexity for multi-state healthcare systems. Commercial insurance dominance generates financial incentives for aggressive treatment; defensive medicine practices lead physicians to err toward intervention rather than palliation. The absence of a unified national AD registry means that documented preferences often remain inaccessible during emergency encounters. In this system, AD completion rates stagnate around 36.7% despite favorable cultural attitudes, and over 35% of hospice patients die within 1 week of enrollment—a pattern consistent with systematic late referrals associated with structural rather than cultural factors.

The United Kingdom maintains high structural resilience through institutional coherence alongside favorable cultural conditions. The unified NHS framework eliminates payment system fragmentation that characterizes American end-of-life care. General practitioners serving as gatekeepers ensure continuous relationships that facilitate advance care conversations. The Gold Standards Framework provides standardized pathways adopted by over 30% of general practices, with research indicating that GSF-accredited teams are twice as likely to enable home deaths (35). The Mental Capacity Act 2005 provides clear statutory grounding for Advance Decisions to Refuse Treatment. The Health and Care Act 2022 further strengthened structural resilience by adding palliative care to the statutory list of services that Integrated Care Boards must commission. This institutional architecture accompanies the United Kingdom’s high end-of-life care quality indicators, including its consistent first-place Quality of Death Index ranking (Table 4).

**Table 4:** Actual levels of resilience from structure (modified by law and institutions).

Country	Impact of legislation (national law)	Impact of payment system (insurance)	Impact of state legitimacy	Actual level
China	Absent; Only Shenzhen local regulation (2023)	No specific palliative care insurance codes; DRG disincentives	Absent; No national agency or endorsement	Low (consistent)
South Korea	Comprehensive: 2018 Life-Sustaining Treatment Act	Full NHI coverage; 354,497 KRW/day inpatient hospice	Strong: National Agency provides legitimation	Higher than potential (dramatic shift)
United States	Fragmented: PSDA federal + 50 state laws	Commercial insurance barriers; defensive medicine incentives	Partial; No unified federal approach	Lower than potential
United Kingdom	Coherent: Mental Capacity Act 2005; Health and Care Act 2022	NHS universal coverage; integrated hospice funding	Strong: NHS framework + GSF implementation	High (consistent)

Institutional indicators and scoring rules for “Actual” Structure are provided in Supplementary Table S1.

**Figure 2:**
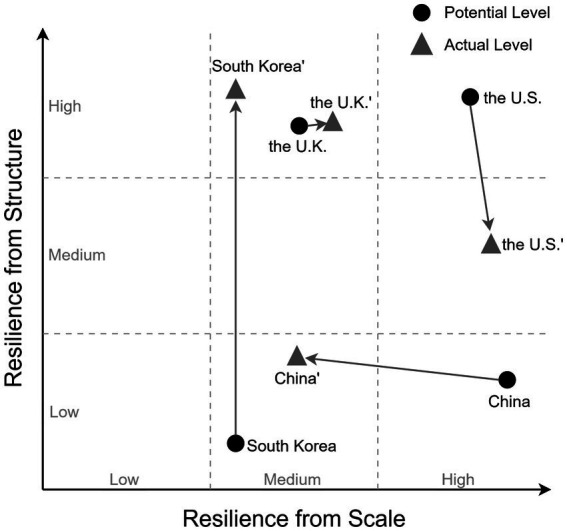
Levels of two types of resilience in four countries (EoL care context). The matrix summarizes four distinct trajectories using ordinal placements. China shifts from (high scale, low structure) to (medium scale, low structure), indicating that substantial resource capacity is underdeployed while structural deficits persist, yielding a downward reclassification in scale with little corresponding change in structure. South Korea shifts from (medium scale, low structure) to (medium scale, high structure), reflecting a prominent upward reclassification in structure that occurred despite culturally unfavorable baselines and without requiring major scale expansion. The United States shifts from (high scale, high structure) to (high scale, medium structure), maintaining resource advantages while experiencing a downward reclassification in structure associated with institutional fragmentation. The United Kingdom remains stable at (medium scale, high structure), reflecting moderate resources that are more consistently converted through institutional coherence. The arrows summarize the direction of each country’s shift from its “potential” to “actual” ordinal placement under the coding rubric (Supplementary Table S1). Rather than quantifying effect sizes, the figure is intended to illustrate the comparative pattern discussed in the text while avoiding metric interpretation. Country placements follow the ordinal coding rubric reported in Supplementary Table S1.

Visualizing resilience shifts: Figure 2 synthesizes the foregoing analysis into a two-dimensional matrix depicting both potential and actual resilience positions for each nation. The horizontal axis represents Resilience from Scale (Low to High); the vertical axis represents Resilience from Structure (Low to High). Each nation is plotted at two positions connected by an arrow: the initial position reflects “potential” levels based on non-political factors; the terminal position reflects “actual” levels after accounting for political and institutional factors. It is important to note that countries at the same level do not imply identical resilience, nor do arrows in the same direction indicate equivalent magnitudes of change. The primary purpose of this figure is to illustrate the relative positioning and directional trajectories of the four countries, rather than to convey precise absolute values.

## 3.2 End-of-life care policies in four countries from the perspective of two types of resilience

To avoid treating the country narratives as stand-alone policy reviews, this section reports each case using a common analytic template aligned with the two resilience dimensions and the coding rubric (Supplementary Table S1). Specifically, the case evidence is summarized only insofar as it informs the institutional “Structure” indicators (legal authority, payment/insurance alignment, and registry/governance legitimation) and the allocation mechanisms that modify realized “Scale” (funding priorities and deployment). The goal is therefore not to provide an exhaustive policy history, but to document the institutional features that justify the ordinal classifications reported in Tables 1–4.

### 3.2.1 End-of-life care policies in China: scale growth, structural lag

China’s end-of-life care development follows a trajectory of expanding Scale capacity accompanied by persistent structural deficits. The following sections document this pattern across three developmental stages. The stages below are used to summarize policy sequencing relevant to the Structure indicators and coding decisions, rather than to provide a comprehensive history of hospice development in China.

Stage One: Philanthropic Introduction (1988–2010). Modern hospice care arrived in China not through government initiative but through private philanthropy (36). The Li Ka Shing Foundation established China’s first hospice unit in November 1988 at Shantou University’s First Affiliated Hospital—a decade after the concept emerged in Western medical systems. The Foundation’s “Heart of Gold” National Hospice Service Program subsequently expanded to over 40 hospice units across 29 provinces, covering more than 350 districts and counties. This philanthropic model—providing free home-based hospice care primarily for terminal cancer patients—has served over 254,000 patients since inception with cumulative investment exceeding RMB 1 billion (approximately $140 million). This approach demonstrated feasibility and generated professional expertise, though it operated at limited scale; charitable resources constituted a small fraction of what systematic government investment could provide in a nation of 1.4 billion people.

Stage Two: Fragmented Local Experimentation (2010–2022). The absence of national policy created space for local innovation, though without mechanisms for systematic replication. Shanghai and Beijing emerged as early pioneers, establishing pilot hospice programs within municipal health systems. However, the landmark development occurred in Shenzhen, which became the first Chinese jurisdiction to legally recognize advance directives when its revised Medical Regulations took effect on January 1, 2023. Article 78 of the Shenzhen Economic Special Zone Medical Regulations stipulates that medical institutions shall respect advance directives for patients in incurable terminal stages, specifying preferences regarding cardiopulmonary resuscitation, tracheal intubation, life support systems, and disease treatment. The regulation requires either notarization or two witnesses not involved in patient care. Shenzhen’s regulation represents the first statutory AD recognition in mainland China, though it operates as a local regulation without national authority. No other Chinese city has enacted similar legislation as of late 2024, and the “Choice and Dignity” platform’s approximately 50,000 national registrations represent a mere 0.005% of the adult population.

Stage Three: National Pilot Expansion (2017-Present). The National Health Commission initiated systematic pilot programs beginning in 2017. The first phase designated 5 pilot cities and districts, including Beijing’s Haidian District, Shanghai’s Putuo District, and cities in Jilin, Henan, and Sichuan provinces. The second phase in 2019 expanded to 71 cities and districts. The third phase in 2023 achieved dramatic expansion to 185 pilot areas including three full provinces (Beijing, Zhejiang, and Hunan) and 61 additional cities, covering 29 of 31 provinces (37). By the end of 2020, 510 hospitals had established hospice care departments. However, this Scale expansion occurred without corresponding structural development; pilot programs operate under general administrative guidance rather than binding legislation, lack dedicated insurance reimbursement codes, and possess no national registration system. Widespread recognition of hospice care’s importance has been accompanied by low actual utilization rates—a phenomenon described in the literature as “applause without attendance” (38).

Structural characteristics: Three interrelated structural features characterize China’s current situation (39). First, the absence of national legislation leaves end-of-life decision-making in legal ambiguity. Physicians report concerns about liability for following patient or family withdrawal preferences without clear statutory protection (40). Families face potential social stigma without official authorization. In this context, there is a systematic default toward aggressive treatment regardless of patient preferences. Second, the payment system creates disincentives for palliative care. Under diagnosis-related group (DRG) payment reforms spreading throughout Chinese hospitals, palliative care generates minimal reimbursement while consuming bed capacity and resources. Public hospital performance evaluation emphasizes surgical volumes, ICU utilization, and revenue generation—metrics under which hospice programs function as lower-priority service lines. Hospital administrators allocate resources toward higher-reimbursement services, with palliative programs receiving limited institutional support. Third, cultural barriers persist in the absence of institutional countermeasures. Without state authorization comparable to South Korea’s National Agency, family members who consent to treatment withdrawal face potential accusations of unfilial behavior (41). The “Choice and Dignity” platform operates without official recognition, meaning that registered preferences carry no legal weight in clinical encounters. These structural features correspond with the current situation in which less than 7% of Chinese patients requiring hospice care currently receive it.

In sum, the China evidence supports the ordinal placements on Scale and Structure summarized in Tables 1–4 (see Supplementary Table S1 for coding rules).

### 3.2.2 End-of-life care policies in South Korea: structure-driven transformation

South Korea’s trajectory is characterized by comprehensive structural intervention following a period of cultural constraints, with substantial increases in AD adoption occurring within a single decade. The stages below summarize the sequencing of institutional changes that are directly relevant to the Structure indicators and the coding rubric, rather than serving as a comprehensive policy chronicle.

Stage One: Traditional Taboo Period (Pre-2008). Prior to external shocks, South Korea exhibited patterns consistent with death avoidance, family-centered decision-making, and absence of patient autonomy in end-of-life contexts. The 1997 Boramae Hospital case crystallized the risks facing physicians who respected family withdrawal preferences: physicians who removed a ventilator at the wife’s request following a patient’s traumatic brain injury were convicted of accessory to murder in 2004. This ruling created a “chilling effect” that persisted for over a decade; physicians adopted defensive practices of continuing aggressive treatment regardless of prognosis to avoid legal liability. Family members, meanwhile, faced difficult choices—either authorize treatment continuation knowing it prolonged suffering, or risk criminal charges and social condemnation for “abandoning” their parent.

Stage Two: Judicial Catalyst and Policy Debate (2008–2016). The “Grandmother Kim” case transformed national discourse. In 2008, a 76-year-old woman entered a persistent vegetative state during a lung biopsy procedure. Her family sought withdrawal of life-sustaining treatment, initiating litigation that reached the Supreme Court. The May 2009 ruling established the principle that medical treatment which “merely extends the dying process without possibility of recovery constitutes a meaningless bodily invasion that harms human dignity.” This decision—widely covered in Korean media—catalyzed public debate about death with dignity and created political space for legislative action. Medical associations issued joint guidelines; the National Bioethics Committee established a Task Force in 2013 that recommended comprehensive legislation. Public opinion, previously resistant to death discussions, shifted toward recognizing the need for legal frameworks governing end-of-life decisions.

Stage Three: Comprehensive Institutionalization (2016-Present). The “Act on Hospice and Palliative Care and Decisions on Life-Sustaining Treatment” passed the National Assembly in January 2016, with hospice provisions taking effect in August 2017 and full enforcement beginning February 4, 2018. A 2020 amendment further expanded covered treatments. The law’s architecture comprises comprehensive structural intervention across multiple dimensions.

Legislative comprehensiveness: The Act establishes clear legal procedures for advance directive registration, specifying required content, witness requirements, and validity conditions. It defines categories of life-sustaining treatments subject to patient decision-making, including cardiopulmonary resuscitation, mechanical ventilation, hemodialysis, and anticancer agents. It provides legal protection for physicians who follow documented patient preferences, addressing the liability exposure that had driven defensive overtreatment since Boramae. The law also incorporates family consensus procedures that permit collective family decisions when patients lack capacity—a mechanism that accommodates Korean familism alongside individual rights frameworks.

Insurance integration: National Health Insurance coverage for hospice services, established in July 2015 prior to full law implementation, ensures financial accessibility. Inpatient hospice receives reimbursement of 354,497 KRW per day (approximately $270), while home-based hospice receives 150,459 KRW per visit (approximately $115). These rates ensure institutional viability; in contrast to China where palliative care functions as a financial liability, Korean hospitals can operate hospice programs as sustainable service lines. Patient out-of-pocket burden decreased substantially following NHI inclusion, expanding access beyond the wealthy (42).

State agency functions: The establishment of the National Agency for Management of Life-Sustaining Treatment constitutes a central structural element. This government agency operates the national AD registry, designates qualified medical institutions (growing from 429 at implementation to 667 by 2024), certifies hospice programs, and provides public education. In addition to these administrative functions, the Agency provides official government endorsement for end-of-life decisions. Its existence creates official state involvement in end-of-life decision-making processes. When an adult child authorizes treatment withdrawal for an aging parent, they do so through government-sanctioned procedures, registered with a national agency, in designated facilities meeting state certification requirements. This official framework provides institutional support for families making end-of-life decisions; the decision occurs within state-sanctioned channels rather than as a purely private family matter.

Outcome evidence: The empirical data show substantial changes following implementation. Cumulative AD registrations grew from fewer than 100,000 in 2018 to 530,000 by 2019 (430% annual growth), 790,000 by 2020, 1.16 million by 2021, 1.57 million by 2022, 2.1 million by 2023, approximately 2.7 million by 2024, and surpassed 3 million in August 2025 (43). Patient self-determination rates—the proportion of end-of-life decisions made by patients rather than families—improved from 32.4% in 2018 to 50.8% by Q4 2024, approaching Western levels despite starting from a Confucian cultural baseline. Hospice utilization among cancer deaths increased from 15% in 2015 to 24.3% by 2019 (44). The number of designated hospice institutions grew from 54 at implementation to 97–100 facilities with 1,546 beds by 2022. These trajectories occurred in temporal correspondence with the institutional interventions described above.

Consistent with the coding rubric (Supplementary Table S1), these institutional features justify South Korea’s placement on both dimensions as reported in Tables 1–4.

### 3.2.3 End-of-life care policies in the United States: high scale, structural friction

The United States combines maximum Scale with structural fragmentation. Unmatched financial investment—$14,000 per capita health expenditure, $23.7 billion Medicare hospice spending, 5,899 certified hospice programs—coexists with systematic inconsistencies that characterize care delivery for many American decedents.

Sequencing overview: U.S. end-of-life policy has evolved through a layered sequence: a federal rights-and-notice framework (PSDA) established baseline expectations for advance directive recognition, while operational implementation has remained largely decentralized across states and provider systems. Over time, the Medicare hospice benefit created a major national payment channel for palliative services, but it has operated within a broader landscape of fragmented commercial insurance incentives. More recently, states and integrated systems have pursued targeted reforms (e.g., POLST and other workflow-oriented tools), producing pockets of coherence within an otherwise patchwork system.

Federal framework without federal coherence: The Patient Self-Determination Act (PSDA) of 1990, effective December 1991, established the first federal requirements for advance directive recognition. The law mandates that Medicare and Medicaid participating healthcare facilities maintain written policies regarding advance directives, inform adult patients of their rights under state law to make medical decisions, document advance directive status in medical records, and provide staff education programs. However, the PSDA deliberately defers substantive regulation to states, creating a federal framework atop 50 different state legal regimes. This fragmentation generates compliance complexity for multi-state healthcare systems, uncertainty for patients crossing state lines, and implementation inconsistency across jurisdictions. Notably, the PSDA does not apply to individual physicians’ offices—a gap that excludes the primary care settings where advance care conversations often occur.

Implementation at the institutional level further affects the operational force of advance directives in the United States. Empirical studies show that, in many hospitals, PSDA compliance is largely procedural: patients are informed of their rights and asked about advance directives during admission, yet the documents are frequently stored as static records rather than integrated into real-time clinical decision-making (45). Advance directives are often inaccessible during emergencies, poorly linked to electronic health records across institutions, or overridden by default life-sustaining protocols in acute care settings. This phenomenon is particularly pronounced in emergency departments and intensive care units, where time pressure and risk aversion favor aggressive intervention over interpretive engagement with prior patient wishes. In this context, the legal recognition of advance directives does not reliably translate into bedside authority, and a gap persists between formal autonomy and clinical practice.

Insurance-related patterns: The commercial insurance dominance of American healthcare generates systematic incentives toward aggressive treatment. Fee-for-service payment models reward intervention volume; physicians and hospitals generate revenue from procedures, imaging, and ICU days, not from hospice referrals or care coordination conversations. Defensive medicine practices—ordering additional tests and treatments to protect against malpractice liability—compound these incentives. In this system, there is a structural default toward overtreatment regardless of patient preferences or clinical appropriateness. While the Medicare hospice benefit represents a notable exception—providing per-diem payments that remove fee-for-service incentives—it covers only the final period of illness and requires physicians to certify six-month prognosis, creating referral hesitancy (46).

Outcome patterns: Structural characteristics are associated with several measurable outcomes. AD completion rates remain around 36.7% of adults with any advance directive and 29.3% with living wills—figures that have remained largely unchanged for decades despite public education campaigns and legal reforms (47). Racial disparities are pronounced: White nursing home residents are three times more likely than Black residents to have living wills (20% versus 6%) and twice as likely to have do-not-resuscitate orders (61% versus 28%) (48–50). Over 35% of Medicare decedents die within 1 week of hospice enrollment, a pattern consistent with systematic late referrals associated with physician reluctance, prognostic uncertainty, or patient/family resistance to “giving up.” The median hospice length of stay of 17 days—versus a mean of 92.1 days reflecting skewed distributions—indicates that many patients receive hospice only in the final days rather than benefiting from its full potential. These outcomes occur in a context of abundant resources, with structural factors representing a relevant consideration.

Heterogeneity and high-performing exceptions: At the same time, the U.S landscape is highly heterogeneous. Several integrated delivery systems and large public providers have implemented more coherent advance care planning workflows (including stronger EHR integration, clinician prompts, and care coordination), and some state-level infrastructures (e.g., mature POLST programs and regional health-information exchange linkages) function as partial substitutes for the absence of a unified national registry. We therefore interpret the U.S classification as a national-level tendency under a common rubric, not as a claim that high-performing subnational or integrated-care models are absent.

State-level innovation: Within the fragmented federal system, states have pursued varied reforms. Physician Orders for Life-Sustaining Treatment (POLST) programs—providing portable, actionable medical orders rather than advisory advance directives—now operate in 48 states (98%), though with varying maturity levels. Medical Aid in Dying (MAID) is authorized in 11 jurisdictions as of November 2025, including Oregon (1997), California, Colorado, and the District of Columbia. These state-level innovations demonstrate structural reform capacity, though the patchwork character characterizes the current landscape. The United States thus exhibits high potential structural resilience based on cultural acceptance of autonomy, combined with reduced actual structural resilience in the context of institutional fragmentation.

This configuration is reflected in the United States’ ordinal classifications reported in Tables 1–4, under the same cross-national coding rules (Supplementary Table S1).

### 3.2.4 End-of-life care policies in the United Kingdom: scale-structure synergy

The United Kingdom combines institutional coherence with moderate resources, achieving high end-of-life care quality indicators. Consistent first-place Quality of Death Index rankings accompany a system characterized by structural coherence that corresponds with available Scale utilization.

Sequencing overview: The UK’s end-of-life governance has developed through a comparatively coherent sequence: statutory grounding for decision-making capacity and advance refusals (Mental Capacity Act), system-level primary-care pathways and quality improvement programs (e.g., GSF), and more recent commissioning reforms that embed palliative care within NHS planning and accountability structures (e.g., Health and Care Act 2022). This sequencing has supported routine clinical normalization of advance care planning through standardized documentation and care pathways.

Unified NHS framework: The National Health Service provides the institutional foundation for structural coherence. Universal coverage eliminates the access barriers and payment system fragmentation seen in American end-of-life care. General practitioners serving as gatekeepers ensure longitudinal patient relationships within which advance care conversations can naturally occur; in contrast to American specialists who encounter patients only during acute episodes, British GPs manage patients across decades, building trust and knowledge that facilitates end-of-life planning. The Health and Care Act 2022 strengthened this framework by adding palliative care to the statutory list of services that Integrated Care Boards must commission—elevating palliative care from discretionary to mandatory system components.

Gold standards framework: The GSF represents a systematic training and accreditation program that standardizes end-of-life care delivery across primary care settings. Developed as a charitable initiative, the GSF has been formally adopted by over 30% of general practices. Research indicates that GSF-accredited teams are twice as likely to care for patients at home until death compared to non-accredited practices. The framework improves identification of patients approaching end of life, enhances communication within multidisciplinary teams, ensures advance care planning conversations occur systematically, and coordinates care across settings. As a national rather than local initiative, the GSF provides standardization in contrast to American state-level variation lacks.

Legal clarity: The Mental Capacity Act 2005 (effective 2007) provides clear statutory grounding for Advance Decisions to Refuse Treatment (ADRT). The Act specifies validity requirements—for life-sustaining treatment refusals, ADRTs must be written, signed, witnessed, and explicitly state applicability “even if life is at risk.” This legal clarity, combined with NHS institutional integration, corresponds with documented preferences being respected in clinical practice. The ReSPECT (Recommended Summary Plan for Emergency Care and Treatment) process further integrates advance care planning into routine clinical documentation, embedding DNACPR decisions within broader emergency care plans. Research indicates that ReSPECT accounts for 52% of the shift from standalone DNACPR forms to comprehensive care planning between 2015 and 2019.

Information and documentation infrastructure: Beyond statutory recognition, the UK places emphasis on standardized, portable care-planning documentation (e.g., ReSPECT) that is designed to be visible and actionable across clinical settings. This operational embedding helps reduce the gap between documented preferences and real-time decision-making, complementing the legal framework and primary-care continuity.

Integrated funding model: UK hospices operate through a unique public-private partnership structure. Total sector operational costs approximate £2 billion annually. Government provides approximately £500 million directly (29–40% of adult hospice costs), with the remaining £1.5 billion derived from charitable giving—fundraising, legacies, and retail operations. This hybrid model creates both advantages and vulnerabilities. The charitable component supplements NHS resources, enabling service levels beyond what tax funding alone could support. However, reliance on donations creates financial sustainability challenges; 59% of children’s hospices ended 2024/25 with deficits, prompting December 2024 government announcements of £100 million capital funding and £26 million children’s hospice revenue support. Despite these pressures, the integrated model is associated with 200 + hospices, 2,570 specialist palliative care beds in charitable hospices (80% of national total), and 630 NHS beds (20%).

Institutional tensions and implementation limits: Despite its high degree of structural coherence, the UK end-of-life care system is not free from internal tensions that constrain full institutional realization. First, regional variation persists within the NHS, particularly in hospice availability, specialist palliative workforce capacity, and the intensity of community-based services, reflecting differences in local commissioning priorities and charitable fundraising capacity. Second, while legal frameworks such as the Mental Capacity Act and procedural tools like ReSPECT provide strong normative guidance, their practical uptake still depends on clinician engagement, time availability, and training, leading to uneven implementation across care settings. Third, the hybrid funding model, while resource-efficient, exposes hospice services to volatility in charitable income, making long-term planning and service expansion vulnerable to macroeconomic fluctuations. These constraints do not negate the UK’s structural advantages, but they underscore that high institutional coherence requires continuous policy maintenance, professional training, and financial stabilization to sustain performance over time.

Outcome indicators: The United Kingdom’s Quality of Death Index score of 93.9 and consistent first-place ranking accompany these structural characteristics. Hospital deaths at 42.8% compare with South Korea’s 75–80% and China’s 90%+. Hospice utilization, GP continuity, and advance care planning integration all exceed international norms. The UK case presents a combination of structural coherence—unified payment systems, standardized care pathways, clear legal frameworks, and integrated funding—alongside high outcome indicators, occurring in a context of moderate Scale.

Together, these elements align with the UK’s high-performing profile on both dimensions in Tables 1–4, using the rubric in Supplementary Table S1.

## 4 Discussion

### 4.1 Summary of findings

This comparative analysis reveals four distinct pathways within the Scale-Structure matrix that illuminate the determinants of end-of-life care system performance. China occupies the lower-left trajectory—high potential Scale reduced by misallocation, combined with persistently low Structure due to legislative and institutional absence. South Korea demonstrates the transformative power of structural intervention—starting from low Structure comparable to China, it achieved dramatic vertical ascent through comprehensive legislation, insurance integration, and state legitimation. The United States occupies an anomalous position—maximum Scale combined with eroding Structure due to fragmentation, producing outcomes inferior to its resource base. The United Kingdom exemplifies optimized efficiency—moderate Scale fully converted through institutional coherence to produce global leadership.

The cross-national patterns observed in the Results section support a structured comparative plausibility interpretation. China’s case is particularly instructive: despite possessing the world’s second-largest aggregate health expenditure, less than 7% of patients requiring hospice care currently receive it. This underperformance reflects not resource scarcity but resource misallocation—a critical distinction with policy implications. Public hospital incentive structures, DRG payment mechanisms, and performance evaluation metrics systematically divert capacity away from palliative care toward more lucrative services. The “Scale without Structure” pattern thus explains why expanding pilot programs and hospice beds without corresponding legislative, insurance, and institutional reform has failed to improve utilization rates. Resources “possessed” are not equivalent to resources “deployed,” and this distinction fundamentally alters how we assess national capacity for end-of-life care.

The United States presents a mirror-image puzzle: maximum resource investment accompanied by suboptimal outcomes. The observed patterns—stagnant AD completion rates around 36.7%, over 35% of hospice patients dying within 1 week of enrollment, and pronounced racial disparities—reflect structural barriers rather than resource limitations. Fifty different state legal frameworks, commercial insurance incentives toward aggressive treatment, and the absence of a unified national AD registry create systematic friction that prevents favorable cultural attitudes toward autonomy from translating into realized care quality. These outcomes are not inevitable consequences of American diversity or federalism; they are products of institutional design choices that could, in principle, be reformed.

The central finding supports comparative plausibility: across the four cases, higher Structural resilience is more consistently aligned with stronger AD institutionalization than Scale alone, while remaining within the inferential limits of an ordinal, theory-building design. South Korea’s trajectory offers the clearest illustration—a nation that transformed from Confucian death taboo to 3 million AD registrations within 7 years through deliberate institutional design. This finding carries profound implications for the cultural determinism that has dominated end-of-life care scholarship. Consistent with our semi-quantitative, theory-building design, we interpret these patterns as comparative plausibility rather than definitive causal identification; the evidence illuminates structured associations and suggests explanatory priorities, but does not constitute formal causal inference.

### 4.2 Theoretical contribution: beyond cultural determinism

A substantial literature has characterized Confucian culture as an inherent barrier to advance directive adoption, emphasizing death taboos, family-centered decision-making, and filial piety as structural impediments rooted in centuries of tradition (51). This cultural determinism has provided intellectual cover for policy inaction—if culture constitutes an immutable constraint, then structural reform becomes futile. The comparative evidence in this study challenges strong versions of this proposition.

The China–South Korea comparison provides a high-leverage within-pair contrast that foregrounds institutional differences under broadly similar cultural conditions. Both nations share a Confucian heritage, including death taboos and familism-inflected norms surrounding end-of-life decision-making. Yet their AD adoption trajectories diverged sharply following South Korea’s post-2018 institutional reforms. If culture were fully determinative at the macro level, the two countries would be expected to exhibit more similar patterns than those observed. This divergence is more consistent with the institutional variables that differ across the pair—comprehensive legislation, insurance integration, and state legitimation in South Korea, versus their absence at the national level in China.

A plausible mechanism involves what we term “state legitimation.” The Korean National Agency for Management of Life-Sustaining Treatment does not merely administer a registry; it provides formal government endorsement and documentation that may alter the moral economy of end-of-life decisions. In Confucian societies, accusations of unfilial behavior can carry substantial social weight—affecting family reputation and social standing. When the state explicitly authorizes and records end-of-life decisions through official channels, family members may receive a form of moral and procedural protection against such accusations. Their decision can be framed as the exercise of state-sanctioned rights within a recognized institutional process rather than as private familial abandonment. This legitimation function—less emphasized in Western analyses that presuppose individual autonomy—offers a plausible cultural-adaptation pathway through which advance care planning can become more institutionally workable in collectivist settings.

The legitimation mechanism operates through multiple channels that deserve elaboration. First, it provides legal certainty: physicians who follow documented patient preferences through state-sanctioned procedures receive statutory protection against liability—addressing the “chilling effect” that persisted since the 1997 Boramae Hospital convictions. Second, it creates procedural formality: decisions made through government-sanctioned channels, registered with a national agency, and executed in designated certified facilities carry official documentation that distinguishes them from informal family choices. Third, it enables moral reframing: what might otherwise be characterized as “abandoning” a parent becomes instead “honoring documented wishes” through legitimate state processes. For adult children navigating the profound obligations of filial piety, this distinction is not merely semantic—it fundamentally alters the social meaning of their actions. This analysis suggests that the “innovative adaptation for Confucian contexts” lies not in modifying the content of advance directives but in embedding them within state-endorsed institutional architectures that provide families with moral cover.

Culture as endogenous and co-evolving with institutions. Importantly, culture in this study is not treated as a fixed external constraint that institutions simply “overcome.” Rather, cultural norms are partially endogenous to legal and policy design: formal rules, official procedures, and state-backed documentation can redefine what counts as morally appropriate family action. In Confucian contexts, “filial piety” is not a single immutable command but a contested moral grammar. When law authorizes advance care planning, routinizes it through registries and clinical workflows, and publicly frames it as protecting patients and families, it can gradually shift social expectations—from “withdrawing care equals abandonment” toward “honoring documented wishes equals responsible care.” As uptake increases, the practice becomes normalized, generating a feedback loop in which institutional implementation further stabilizes new norms, which in turn reinforces policy effectiveness.

### 4.3 Practical implications: a structural adaptation model

These findings generate a “Structural Adaptation Model” offering actionable guidance for developing nations—particularly those with cultural contexts that challenge Western autonomy paradigms. The model comprises three priority interventions. First, nations should pursue national-level legislation that provides legal certainty for both patients and providers, incorporating culturally appropriate provisions for family proxy decision-making that reconcile individual autonomy with collectivist values. Second, dedicated health insurance codes and reimbursement structures for palliative care are essential; without financial viability, hospice programs remain charitable endeavors dependent on philanthropic subsidy. Third, national registration agencies should be established not merely for administrative efficiency but for the legitimation function they perform—providing state endorsement that enables culturally constrained families to exercise end-of-life decision-making rights.

Norm-shaping role of legal frameworks: Legal frameworks do not only provide enforcement and procedural clarity; they also function as norm entrepreneurs. By specifying default options, roles, and documentation standards, and by signaling official endorsement, law can make end-of-life planning socially “sayable” and professionally “doable.” Over time, repeated clinical use (e.g., routine prompting, standardized forms, registry visibility, and professional training) helps translate abstract rights into ordinary expectations. This norm change then feeds back into higher participation and stronger implementation, creating a self-reinforcing cycle of institutionalization → normalization → increased adoption.

The United Kingdom case offers additional insights for nations with higher resource bases. The UK demonstrates that structural coherence—unified payment systems, standardized care pathways, clear legal frameworks, and integrated funding—can compensate for moderate Scale to produce exceptional outcomes. This finding carries particular relevance for the United States, which possesses substantially greater resources but achieves inferior results. The UK’s institutional architecture suggests that consolidating fragmented systems, standardizing care protocols (as the Gold Standards Framework has done), and integrating funding streams may yield greater returns than additional resource investment in a structurally incoherent system. For nations with expanding Scale, such as China, the UK case suggests that building institutional coherence concurrently with resource expansion—rather than sequentially—would optimize the translation of inputs into outcomes. The contrast between UK and US outcomes, despite the latter’s tenfold greater per capita health expenditure, underscores that structural design choices matter more than absolute resource levels in determining end-of-life care quality.

For China specifically, the Shenzhen experiment provides a foundation but requires national extension. The current pilot approach—expanding hospice bed capacity without structural reform—reproduces the “Scale without Structure” pattern that produces underperformance. Prioritizing structural reform over further Scale expansion would yield higher returns; South Korea achieved its transformation with moderate resources by channeling them through effective institutional conduits. For the United States, the lesson is structural coherence—consolidating fragmented state systems, aligning payment incentives toward appropriate care, and establishing national AD portability mechanisms.

Boundary conditions and likely failure modes: The Structural Adaptation Model is most likely to underperform when minimum enabling conditions are absent. In low-capacity settings, legislation can remain largely symbolic if there is inadequate palliative workforce, limited access to essential medicines, weak primary-care continuity, or insufficient administrative capability to operationalize documentation and referral pathways. In highly fragmented healthcare systems, the model can also fail through incentive mismatch: even when legal rights exist, decentralized payers and providers may lack aligned reimbursement, shared accountability, or interoperable documentation, resulting in persistent late referrals and default aggressive treatment. Finally, implementation can be undermined when state legitimation is weak or contested—if patients and families do not trust official registries or fear social and institutional consequences, documentation may not translate into clinical use.

Mitigation strategies and sequencing: To address these risks, the model should be implemented as a staged reform package rather than as a single-step institutional transplant. For low-capacity settings, a “minimum viable” pathway prioritizes (i) basic service capability (training and task-sharing for frontline clinicians, essential medicine access, and community-based palliative delivery), followed by (ii) narrow but enforceable legal defaults and standardized documentation, and only then (iii) scaled registry infrastructure. For fragmented systems, reform should focus on creating functional coherence: adopting common portability standards for AD/ACP documentation, aligning payment incentives across major payers (e.g., bundled or per-diem models that reward coordination), and embedding prompts and visibility into routine workflows so that documents are actionable in emergencies. In all contexts, monitoring should explicitly separate intermediate implementation outputs (registration/coverage) from downstream outcomes (timing of hospice, place-of-death patterns, and quality-of-death measures), so that policy learning can identify which link in the conversion chain is failing and target corrective adjustments.

### 4.4 Limitations

Several limitations warrant acknowledgment. Data comparability challenges arise from differing national definitions of “palliative care beds,” “hospice services,” and “advance directives” that complicate cross-national comparison. Second, AD registration is an intermediate policy output and does not guarantee preference-concordant treatment at the bedside. Cross-nationally comparable measures of concordance and patient-reported end-of-life experience are limited; accordingly, we use AD registration as a proxy for institutionalization and triangulate it with downstream outcome indicators rather than equating registration with care quality. Third, the Structural Adaptation Model presumes a minimum level of service and administrative capacity; in low-capacity or highly fragmented systems, institutional reforms may not translate into practice without phased implementation and complementary capacity-building. The temporal scope of 2019–2024 captures substantial change in South Korea and China but may miss longer-term trends in the United States and United Kingdom. The COVID-19 pandemic disrupted end-of-life care systems globally, potentially introducing confounding factors not fully addressed. Finally, our four-case design—while theoretically motivated—limits generalizability to nations beyond the selected sample. Future research should expand to additional Asian cases (Japan, Taiwan, Singapore) and test the Structural Adaptation Model through implementation studies.

## 5 Conclusion

As humanity confronts the “silver tsunami” of global aging, ensuring dignified death becomes an imperative transcending national boundaries. This study suggests that such dignity depends not on cultural fortune or resource abundance but on deliberate institutional design. South Korea’s transformation from Confucian constraint to structural resilience illuminates a path for other Asian nations; the United Kingdom’s coherent framework shows how modest resources can achieve global leadership; the United States’ fragmentation warns against assuming that cultural acceptance suffices without institutional support; and China’s current trajectory reveals the costs of Scale without Structure. The challenge for policymakers is clear: invest not merely in beds and physicians but in the legal, financial, and administrative architecture that converts resources into care (52). In the architecture of dying well, structure is destiny.

## Data Availability

Publicly available datasets were analyzed in this study. This data can be found here: Publicly available datasets were analyzed in this study. The data sources are available at: OECD Health Statistics: https://stats.oecd.org/ WHO Global Health Observatory: https://www.who.int/data/gho; Korea National Agency for Management of Life-Sustaining Treatment: https://www.lst.go.kr/eng/index.do; U.S Centers for Medicare & Medicaid Services (CMS): https://data.cms.gov/ NHS England Statistics: https://www.england.nhs.uk/statistics/ National Health Commission of the PRC: http://www.nhc.gov.cn/ World Bank Open Data: https://data.worldbank.org/.
