# Supplementary material for: From cultural constraints to structural resilience: a comparative analysis of end-of-life care policies in China, South Korea, the U.S., and the U.K
Source: Front Public Health. 2026 Mar 3;14:1756405. doi: 10.3389/fpubh.2026.1756405 (PMC12992241; doi:10.3389/fpubh.2026.1756405)
Supplement: Supplementary file 1 [file Data_Sheet_1.pdf]

# Table S1. Coding Rules for Cross-National Comparison of End-of-Life Care Resilience

| Dimension | Sub-dimension | Indicator (as used in manuscript) | Coding Rule (High=2 / Medium=1 / Low=0) | Role | Main Data Source & Year |
|---|---|---|---|---|---|
| **Scale (Potential)** | Demographic | Older population (65+) absolute number | High (2): ≥50 million<br>Medium (1): 10–49.9 million<br>Low (0): <10 million | Potential; Measure | World Bank / National Statistics (2023) |
| | Economic | Per capita health expenditure (PPP); Total health expenditure global rank | High (2): Per capita ≥$10,000 (PPP) OR total spending globally top-3<br>Medium (1): Per capita $3,000–9,999 (no top-tier total)<br>Low (0): Per capita <$3,000 (no top-tier total) | Potential; Measure | OECD Health Statistics; WHO GHED (2022–2023) |
| | Medical Resource | Physician workforce total; Palliative care beds per million; PC specialist workforce | High (2): Physician workforce ≥1,000,000 OR PC bed density ≥70/million OR large PC specialist workforce (≥15,000)<br>Medium (1): Physician workforce 200,000–999,999 OR bed density 20–69/million<br>Low (0): Bed density <20/million AND physician workforce <200,000 | Potential; Measure | WHO; Global Atlas of Palliative Care; NHPCO (2020–2024) |
| | **Aggregation Rule** | Overall Potential Scale category | High: ≥2 sub-dimensions coded High<br>Medium: <2 High AND 0 Low (all Medium or 1 High + 2 Medium)<br>Low: ≥2 sub-dimensions coded Low | Potential; Derived | — |
| **Scale (Actual)** | Funding Priorities | Dedicated palliative care budget lines; Government expansion programs | High (2): Dedicated budget lines + active expansion programs<br>Medium (1): General health funding without specific PC allocation<br>Low (0): No dedicated PC funding; competing priorities | Actual; Proxy | National policy documents; Ministry reports (2019–2024) |
| | Resource Allocation | Curative vs. palliative balance; Hospital incentive structures (DRG impact) | High (2): Balanced allocation OR palliative-supportive incentives<br>Medium (1): Partial skew toward curative; some PC support<br>Low (0): Heavily skewed toward curative/acute care; DRG disincentives for PC | Actual; Proxy | Health system analyses; payment reform documents (2019–2024) |
| | **Determination Rule** | Actual Scale level (modified from Potential) | Start from Potential Scale level, then adjust:<br>• If both policy indicators = 0: Actual will drop one level<br>• If both policy indicators = 2: Actual will rise one level or confirm High<br>• Mixed scores (0+1, 1+1, 1+2): Actual = Potential (no change) | Actual; Derived | — |
| **Structure (Potential)** | Cultural Factors | Death taboo prevalence; Family-centered vs. individual decision-making tradition; Filial piety obligations | High (2): Individualist autonomy culture; death-positive movement; patient as primary decision-maker<br>Medium (1): Mixed/transitional; partial autonomy acceptance<br>Low (0): Strong death taboo; family-centered decisions; Confucian filial piety dominant | Potential; Proxy | Cultural studies; ACP literature (cross-sectional) |
| | Digital Infrastructure | Internet penetration rate; Mobile connectivity; E-health readiness | High (2): Internet penetration >70%; robust e-health systems<br>Medium (1): Internet penetration 50–70%<br>Low (0): Internet penetration <50% | Potential; Measure | ITU; World Bank Digital Development (2022–2023) |
| | Public Awareness/ Education | Health literacy levels; Public campaigns on ACP; Educational attainment | High (2): High health literacy; active public ACP campaigns<br>Medium (1): Moderate literacy; limited campaigns<br>Low (0): Low health literacy; minimal ACP awareness | Potential; Proxy | WHO; National surveys (2019–2023) |
| | **Aggregation Rule** | Overall Potential Structure category | Cultural constraint is treated as a baseline limitation but not a deterministic veto: | Potential; Derived | — |

| Dimension | Sub-dimension | Indicator (as used in manuscript) | Coding Rule (High=2 / Medium=1 / Low=0) | Role | Main Data Source & Year |
|---|---|---|---|---|---|
| | | | • If Cultural = High (2) → Potential Structure = High<br>• If Cultural = Medium (1) → Potential Structure = the rounded average of the three sub-dimensions (Culture, Digital, Awareness)<br>• If Cultural = Low (0):<br>– If Digital = High (2) and Awareness/Education = High (2) → Potential Structure = Low/Medium<br>– Otherwise → Potential Structure = Low | | |
| **Structure (Actual)** | Legislation | National EoL/AD law; Legal authority and scope; Physician liability protection | High (2): Comprehensive national law with clear legal authority + liability protection (e.g., Korea 2018 Act, UK MCA 2005)<br>Medium (1): Partial/fragmented—federal baseline + state variation OR incomplete scope (e.g., US PSDA + 50 state laws)<br>Low (0): Absent—no national law; only local pilots or none (e.g., China—only Shenzhen 2023) | Actual; Proxy | National legislation texts; Legal databases (current as of 2024) |
| | Payment Alignment | National insurance PC coverage; Reimbursement rates; Incentive alignment | High (2): National insurance explicitly reimburses hospice/palliative with stable incentives (e.g., Korea NHI 354,497 KRW/day; UK NHS universal)<br>Medium (1): Partial coverage OR misaligned incentives (e.g., US Medicare hospice benefit but commercial insurance barriers)<br>Low (0): No meaningful coverage; no specific PC codes; DRG disincentives (e.g., China) | Actual; Proxy | NHI/NHS/Medicare policy documents (2022–2024) |
| | State Legitimation | National registry/agency; Official endorsement; Implementation authority | High (2): National agency with official endorsement + implementation authority (e.g., Korea NALST; UK NHS/GSF framework)<br>Medium (1): Partial/indirect—no unified federal approach but some state-level systems (e.g., US POLST programs)<br>Low (0): Absent—no national agency or endorsement; NGO platforms only (e.g., China 'Choice and Dignity') | Actual; Proxy | Government agency reports; Registry data (2018–2024) |
| | **Aggregation Rule** | Overall Actual Structure category | Sum scores of Legislation + Payment + State Legitimation (range 0–6):<br>• Sum 5–6 → High<br>• Sum 3–4 → Medium<br>• Sum 0–2 → Low | Actual; Derived | — |

**Notes:**

• Role column: 'Potential' = non-political baseline capacity/constraints; 'Actual' = post-policy implementation level.

• Role column: 'Measure' = indicators based on internationally harmonized quantitative data; 'Proxy' = institutional features with explicit rule-coded criteria; 'Derived' = aggregated from sub-dimension scores.

• PPP = Purchasing Power Parity; PC = Palliative Care; AD = Advance Directive; EoL = End-of-Life; NHI = National Health Insurance; NHS = National Health Service; DRG = Diagnosis-Related Group.

• NALST = National Agency for Management of Life-Sustaining Treatment (Korea); GSF = Gold Standards Framework (UK); POLST = Physician Orders for Life-Sustaining Treatment; PSDA = Patient Self-Determination Act; MCA = Mental Capacity Act.

• Data sources: OECD = Organisation for Economic Co-operation and Development; WHO = World Health Organization; GHED = Global Health Expenditure Database; ITU = International Telecommunication Union; NHPCO = National Hospice and Palliative Care Organization.

• Year alignment. Unless otherwise noted, we use the most recent observation available within the study window (2019–2024) and, where feasible, align countries around a common reference period (typically 2021–2023). Country-specific years are reported in the “Source & year window” column; when exact year alignment is not feasible, indicators are interpreted ordinally rather than as exact point estimates.

• Comparability and indicator status. Internationally harmonized series (OECD/WHO/World Bank) are treated as formal quantitative measures. Country-specific administrative counts (e.g., registry-based AD registrations; hospice/palliative capacity reported by national agencies or professional bodies) are treated as rule-coded indicators (proxies), and comparisons are interpreted with

attention to national definitional scope and reporting practices.

• Inference and triangulation. For indicators with partial cross-national comparability (e.g., “hospice services,” “palliative care beds,” and the operational meaning of “advance directives”), classification relies on triangulation across multiple sources and on low/medium/high ordinal placement rather than assumptions of numerical equivalence.

## Table S2. MSSD Implementation Matrix and Rival-Explanation Checks (Four-Country Set)

| Comparison block (nested pairs) | Matching conditions (treated as broadly similar / held constant for within-pair inference) | Key institutional contrasts examined (Structure mechanisms) | Primary rival explanations assessed | Expected pattern if rival explanation holds | Observed pattern in this study (as summarized in main text tables/figure) | Inference logic (what this block rules out / supports) |
|---|---|---|---|---|---|---|
| **East Asian pairing (MSSD core): China vs South Korea** | Shared Confucian cultural heritage (filial piety, family-centered decision-making, death taboo); rapid development trajectory (agrarian→industrial within a generation); high digital readiness enabling national registration systems | National legal authority: comprehensive national statute vs fragmented/local regulation; insurance/payment alignment: full National Health Insurance (NHI) integration vs absent/limited coverage; state legitimation and implementation authority: dedicated national agency/official endorsement and registry governance vs lack of unified national mandate/endorsement | Cultural determinism (Confucian societies inherently resist ACP/AD); technology sufficiency (digital readiness alone drives uptake); resource endowment as sufficient driver | If culture is determinative → both countries should remain similarly low in AD implementation; if technology is sufficient → both should show similarly high implementation given robust digital infrastructure; if resources are sufficient → higher resource endowment should align with better outcomes regardless of institutions | Divergence within shared Confucian constraints: South Korea shows rapid institutional "take-off" after comprehensive reform and NHI alignment, while China remains low-uptake under fragmented rules and weak institutional conversion capacity | Weakens "culture/technology as sufficient explanations"; supports Structure (institutional conversion capacity) as the more plausible driver of divergence under similar cultural constraints |
| **Western pairing (institutional contrast within broadly similar value tradition): United States vs United Kingdom** | Western individualist tradition emphasizing patient autonomy; mature EoL sectors with established hospice/palliative care; high-income setting with professionalized health systems and widespread digital infrastructure | Financing/coordination regime: market-dominated commercial insurance + fee-for-service + defensive medicine vs unified NHS commissioning and system integration; legal coherence: federal baseline plus 50-state variation vs coherent statutory grounding (e.g., Mental Capacity Act) and standardized pathways; information/standardization infrastructure: fragmented documentation/registry access vs more coherent primary-care gatekeeping and standardized care pathways (e.g., GSF) | Resource abundance as sufficient driver (more spending/resources → better EoL outcomes); autonomy culture as sufficient driver (shared individualist norms → similar uptake/outcomes) | If resources are sufficient → US should outperform UK given much higher spending; if autonomy culture is sufficient → both should perform similarly high | Paradox pattern: US shows high Scale yet weaker realized coordination and late hospice timing (fragmentation); UK achieves benchmark performance through institutional coherence despite more modest resources | Weakens "resources/autonomy as sufficient explanations"; supports institutional coherence, payment alignment, and care coordination as key conversion mechanisms |
| **Cross-group plausibility check (East Asia vs West): (China & South Korea) vs (US & UK)** | Designed as a cross-pair test of boundary conditions: culture differs across region-pairs while "Structure vs Scale" varies within each pair | Tests whether institutional intervention can overcome cultural constraints, and whether favorable culture can still underperform under fragmentation; also checks whether the framework's Structure mechanism explains mixed regional patterns better than "East vs West" dichotomies | Pure cultural barrier claim (Confucian context blocks AD regardless of institutions); pure affluence claim (high-income settings uniformly achieve high outcomes) | If culture is an absolute barrier → both China & Korea should remain uniformly low vs West uniformly high; if affluence is decisive → US & UK uniformly high and superior to East Asian cases | Mixed pattern: Korea approaches Western-level uptake after structural intervention (despite Confucian context), while US underperforms relative to UK due to fragmentation (despite autonomy culture and high spending) | Supports the paper's central claim that institutions mediate the relationship between culture/resources and outcomes; positions evidence as comparative plausibility rather than definitive causal identification |
| **Two-dimensional synthesis (framework-** | Uses the same four-case set but treats it as a two-dimensional comparative | Conversion efficiency logic: Scale provides the resource substrate; Structure determines conversion into | Post hoc classification concern (placements are narrative rather than rule- | If placements are post hoc → readers cannot reproduce the mapping; if | Rule-based ordinal coding now specified (Table S1) + | Directly answers reproducibility critique by linking (i) explicit coding |

| Comparison block (nested pairs) | Matching conditions (treated as broadly similar / held constant for within-pair inference) | Key institutional contrasts examined (Structure mechanisms) | Primary rival explanations assessed | Expected pattern if rival explanation holds | Observed pattern in this study (as summarized in main text tables/figure) | Inference logic (what this block rules out / supports) |
|---|---|---|---|---|---|---|
| **consistency check): Scale × Structure positioning (potential vs actual)** | space; emphasizes ordinal placements rather than precise measurement | accessible, coordinated, preference-concordant services; contrasts "potential" vs "actual" levels after policy implementation | based); single-factor dominance (Scale alone or culture alone) | single-factor dominance → one axis should explain outcomes without needing the other | potential/actual shifts displayed (Fig 2); countries show different trajectories consistent with "conversion efficiency" logic | rules and (ii) an interpretable synthesis map; strengthens methodological transparency and shows how inference is structured |

**Notes**:

- "Matching conditions" are treated as approximate similarities that increase design leverage for within-pair inference; they do not imply full equivalence on all background variables.
- "Observed pattern" statements summarize the manuscript's comparative findings as reported in Tables 1–4 and Figure 2; the design supports comparative plausibility rather than definitive causal identification.

## Table S3. Analytical Dimensions, Indicators, and Their Methodological Roles

| Analytical Dimension | Indicator | Data Source / Type | Inclusion Criterion | Methodological Status | Analytical Role |
|---|---|---|---|---|---|
| **Scale** | Per-capita health expenditure (PPP) | OECD / WHO harmonized statistics | Internationally standardized definition; cross-national comparability | Formal quantitative measure | Anchors relative resource intensity across cases; used for ordinal cross-country comparison |
| **Scale** | Public and private spending on hospice / palliative care | OECD; national health accounts | Availability of sector-level expenditure data | Formal–proxy hybrid | Indicates financial commitment to end-of-life care within overall health spending |
| **Scale** | Hospice and palliative care service capacity (institutions, beds) | National registries; government or professional bodies | Nationally reported administrative data | Rule-coded indicator (proxy) | Supports ordinal classification (low/medium/high) of service availability |
| **Scale** | Trained palliative care workforce density | National statistical agencies; professional associations | Recognized specialty or certified training | Rule-coded indicator (proxy) | Contextualizes capacity constraints and delivery potential |
| **Structure** | Legal recognition of advance directives / ADRTs | Statutory law and binding regulations | Explicit legal validity and enforceability criteria | Typological legal indicator | Assesses formal institutional clarity and normative authority |
| **Structure** | Institutional integration of advance care planning | Health system design; NHS / hospital governance rules | Integration into routine clinical workflows | Heuristic institutional indicator | Captures degree of operational embedding beyond legal recognition |
| **Structure** | Registry and documentation governance (e.g., national or interoperable registries) | Administrative rules; system architecture | National or regional scope with clinical accessibility | Rule-coded indicator (proxy) | Evaluates conversion capacity from documented wishes to bedside action |
| **Structure** | Payment and incentive alignment | Insurance design; reimbursement rules | Presence of incentives for ACP and palliative referral | Analytical construct (synthetic) | Explains structural facilitation or distortion of end-of-life decision-making |
| **Structure** | Standardized clinical pathways (e.g., GSF, POLST, ReSPECT) | National programs and guidelines | Formal adoption and routine use | Heuristic programmatic indicator | Signals system-level coherence and implementation consistency |
| **Cross-cutting** | Outcome alignment (place of death, timing of hospice entry) | OECD; national mortality statistics | Comparable outcome definitions | Interpretive outcome indicator | Used for plausibility checks rather than causal validation |
